# Pacific oysters (*Magallana gigas,* Thunberg 1793) preferentially consume *Isochrysis galbana,* increasing biomass and upregulating biomineralisation gene nacrein

**DOI:** 10.1007/s10499-025-02161-y

**Published:** 2025-10-03

**Authors:** Amy Lovegrove, Sargent Bray, Gordon Inglis, Megan Wilding, Bastian Hambach, Chris Hauton

**Affiliations:** 1 Faculty of Environment and Life Science, School of Biological Sciences, University of Southampton, Southampton, SO17 1BJ UK; 2 Present Address: Faculty of Environment and Life Science, School of Ocean and Earth Science, University of Southampton, Southampton, SO14 3ZH UK; 3 Centre for Environment Fisheries and Aquaculture Science, Pakefield Road, Lowestoft, NR33 0HT UK

**Keywords:** Pacific oyster, Aquaculture, Biomineralisation, Shellfish, Microalgae, Molecular biology, Stable isotopes, Feeding experiment

## Abstract

Microalgae are the foundation of oyster diets in aquaculture. As demand for oysters increases, so does the need for nutritionally complete diets. *Isochrysis galbana* is considered the optimal oyster diet and is often supplemented with other algae like *Nannochloropsis* to provide complementary nutrients, but which diet do the oysters prefer, and what effects do the diets have on physiology? This study performed feeding experiments with single (*I. galbana* or *Nannochloropsis*) and mixed (both genera combined) diets in Pacific oysters (*Magallana gigas*). Oysters fed exclusively *I. galbana* had greater biomass gains but reduced shell growth, evidenced by a lower Oyster Condition Index, and gene expression analysis showed compensatory upregulation of the biomineralisation gene nacrein in this group*.* Oysters fed mixed diets showed higher algal cell clearance and pseudofaeces production, and within the mixed diet, a preference for *I. galbana*. This suggests that whilst a mixed-algae diet is traditionally used, a single species diet of *I. galbana* can significantly enhance oyster growth, reducing the need for complex multi-species algal culture. Culturing one alga is more time- and cost-effective, but stage-specific diets could promote specific physiological factors. These findings can help to optimise oyster feeding in a world with increasing demand for oysters.

## Introduction

Pacific oyster (*Magallana gigas*) (Thunberg, 1793) aquaculture is a thriving industry crucial to global seafood production, with an estimated global value of $23.9 billion in 2017 (van der Schatte Olivier et al. 2020). China leads in oyster production and consumption, accounting for 78% of the production value and 86% of the production weight (Botta et al. 2020). Although this includes various oyster species, *M. gigas* and the Suminoe oyster (*Crassostrea ariakensis*) are the primary cultivated species (Li and Mori 2006). As of 2021, other major *M. gigas*-producing countries include Korea (14.6 million tonnes year^−1^), India (305,914 tonnes year^−1^), Japan (158,400 tonnes year^−1^), France (85,170 tonnes year^−1^), and the USA (21,312 tonnes year^−1^) (OECD Stat).

A key factor contributing to the success of oyster farming is the careful management of oyster nutrition, where microalgae serve as essential dietary components that sustain oyster health and growth. In farms, Pacific oysters are primarily fed the haptophyte *Isochrysis galbana*, considered the ‘gold standard’ of bivalve feed due to its high concentrations of fucoxanthin and docosahexaenoic acid (DHA, 22:6, n-3) (Poisson and Ergan 2001; Liu et al. 2013), and its palatable size and shape (Islam et al. 2025). Whilst species from the *Nannochloropsis* genus are also widely used for their rich eicosapentaenoic acid (EPA, 20:5, n-3) content (Sukenik 1991; Sá et al. 2020*), I. galbana* remains superior in overall nutritional value for bivalves (Pereira et al. 2023). The optimal feed in oyster aquaculture, *I. galbana*, provides high ω−3 PUFAs like EPA and DHA, carotenoids, and fucoxanthin, which are essential for the growth and survival of oyster larvae and juveniles (Bhattacharjya et al. 2020; Kumar et al. 2023). Its nutritional composition of 12%–14% lipids, 50%–56% proteins, and 10%–17% carbohydrates has been shown to be the best configuration of nutrients for oyster development (Milledge 2011; Wikfors et al. 1992).

Considerable research has focused on evaluating bivalve diets in aquaculture (e.g. Knauer and Southgate 1999 and references therein), claiming that multi-species diets (e.g. *Chaetoceros* *calcitrans, Dunaliella tertiolecta, Isochrysis* sp. strain T. ISO, *Rhodomonas salina*, and *Skeletonema costatum*) significantly enhance oyster growth rates and maintain higher levels of EPA and DHA compared to single-species diets (McCausland et al. 1999; Guedes and Malcata 2012). The argument is that a mixed species microalgal diet provides a comprehensive nutrient profile that a single-species diet could not supply, offering the best chance for oyster survival (Brown et al. 1998; Spolaore et al. 2006; Marshall et al. 2010).

However, studies on the physiological effects of mixed-species diets have yet to determine whether oysters ingest cells of different algal species at equal rates. Variations in growth rate, survival, and metamorphosis may occur depending on the specific species and nutrients consumed more frequently. Previous research on selective feeding mainly used light microscopy, cell density calculations, and indicator pigments to track microalgae species throughout the digestive system (e.g. Rahman et al. 2020; Jiang et al. 2022; Weissberger and Glibert 2021). These approaches, whilst useful, face challenges in quantifying exact densities of different microalgae cells, especially relative to other species present in the system.

One specific aspect of oyster physiology linked to diet is shell formation. Pacific oysters construct their shells using calcium carbonate (CaCO_3_) from their environment (McDougall and Degnan 2018), and studies have shown that diets rich in ω−3 and ω−6 fatty acids significantly enhance shell formation, as evidenced by decreased meat weight and visible shell growth (Trider and Castell 1980; Kniprath 1980; Weiss et al. 2002; Marin and Luquet 2004).

Investigating the effect of different microalgal diets on the shell formation of Pacific oysters involves understanding how these diets influence the expression of key genes involved in biomineralisation. Calmodulin (*Cg_CaM*) encodes a calcium-binding messenger protein involved in calcium signalling pathways through regulating calcium-dependent enzymes and structural proteins (Li et al. 2004). Mantle gene 4 (*Cg_MG4*) is involved in the formation of the organic matrix of the shell, which serves as a scaffold for the deposition of calcium carbonate crystals (Liu et al. 2007). Perlucin (*Cg_Perl*) is involved in the nucleation and growth of calcium carbonate crystals in the initial stages of shell formation by promoting the crystallisation process (Mann et al. 2000), and so monitoring the expression of perlucin helps in understanding how dietary variations impact the early stages of shell biomineralisation and crystal formation. Nacrein-like protein F2 (*Cg_Nac*) has carbonic anhydrase-like domains that facilitate the conversion of CO_2_ to bicarbonate (HCO_3_^−^) which is used in the regulation of aragonite and calcite crystallisation (Song et al. 2014). By using RT-qPCR to measure the expression levels of these genes, the molecular mechanisms underlying shell formation in response to different microalgal diets can be better understood. This approach allows for the identification of specific dietary components that enhance or inhibit shell growth, contributing to optimised feeding strategies for sustainable aquaculture.

Stable isotopes (SI) δ^15^N and δ^13^C are commonly used as bioindicators for diet analysis and quantifying organic contributions throughout food chains (Skinner et al. 2022 and references therein); specifically, ^15^N is a heavy isotope of nitrogen, a vital plant nutrient used to quantify nitrogen fixation (McClelland et al. 2003; Cox et al. 2022). Nitrogen stable isotopes are powerful predictors of trophic structures, as they accumulate through the food chain, resulting in quantifiable increases in δ^15^N (Steinkopf et al. 2024) that can be measured to identify individual species of macroalgae due to different metabolic pathways influencing δ^15^N uptake (Duarte et al. 2018). In *I. galbana,* the culture media nutrient composition has been shown to influence isotopic fractionation, which led to species-specific SI signatures (Camacho-Rodríguez et al. 2021).

This study aimed to investigate the relationship between Pacific oyster diet microalgae species and oyster physiology, specifically focusing on the ratio of *I. galbana* and *Nannochloropsis* cells consumed when offered a mix of both. We examined respiration rates, changes in total mass, Oyster Condition Index (OCI), algal cell clearance rates, the mass of rejected algal cells, the SI composition of the diet microalgae and oyster gut contents, and finally, the expression of four target biomineralisation genes with each diet condition. The principal objective was to assess the effect of *I. galbana* and *Nannochloropsis* spp., independently and as a mixed diet, on these physiological parameters following an 11-week feeding experiment from December 15th 2021 to February 25th 2022.

## Methods

### Feeding trial

**Fig. 1:**
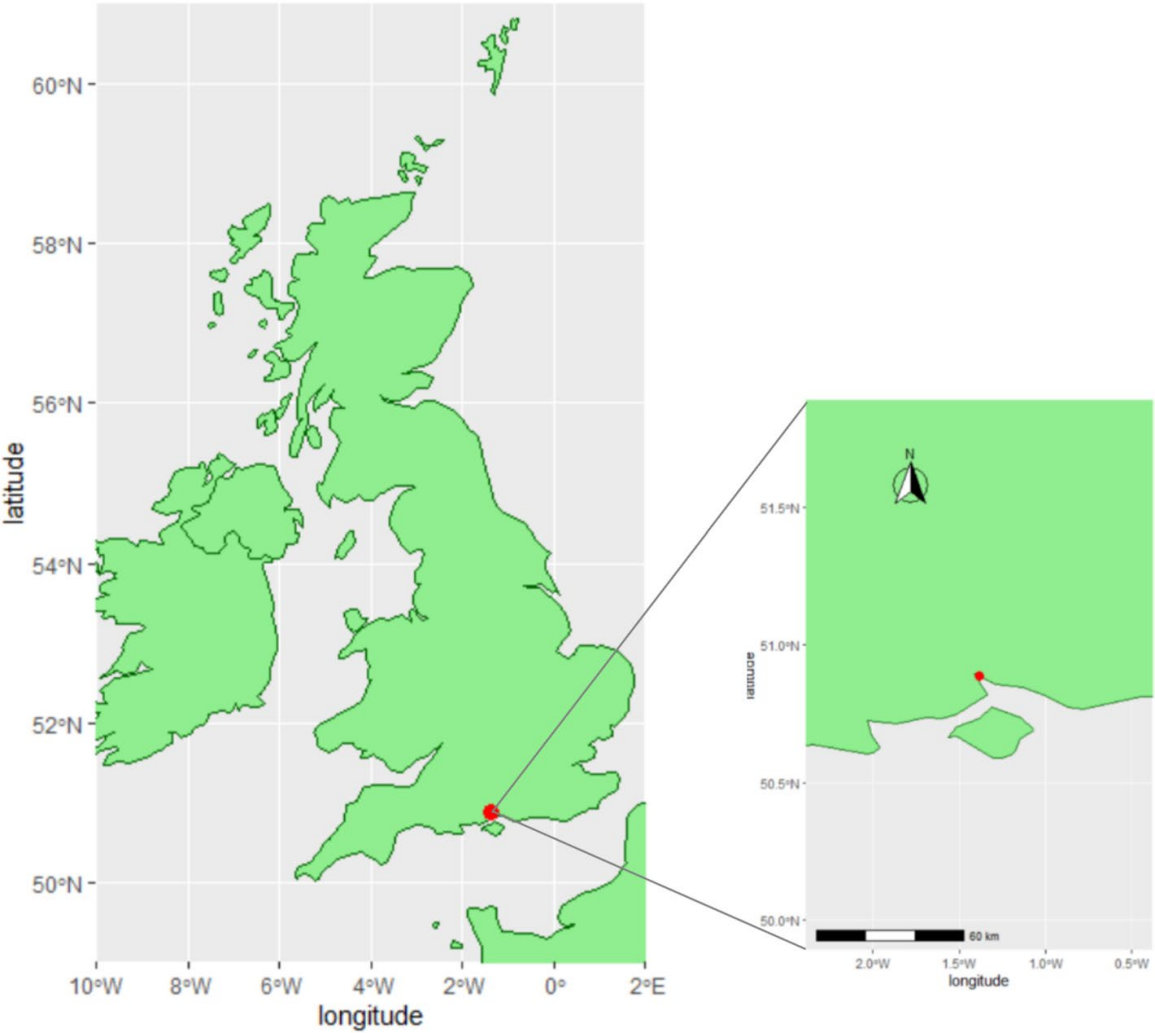
Map of the UK with coordinates of oyster sampling site shown in red, and a zoomed perspective of the Southampton coast

Ethical approval for this work was obtained under the Ethics Research and Governance Online from the University of Southampton (ERGO II) under reference 67763. Seventy-two wild, adult *Magallana gigas* were collected from the Weston Shore Promenade (50° 53′ 21.9″ N 1° 22′ 59.0″ W; Fig. 1) at the mouth of the River Itchen into Southampton Water at low tide in October 2021. Oysters were acclimated for two weeks in a 400 L tank in the University of Southampton aquarium and fed periodically.

After initial wet mass and total valve length (TVL) measurements were taken, the oysters were moved to a 1200 L tank where they were separated into three feeding groups of 24. Six oysters were kept in each 8 L container, with four containers per diet condition (*I. galbana* (Iso), *Nannochloropsis* spp. (Nan), and mixed diet (Mix)). The oysters were moved into the main flow-through tank (324 L filled volume). Oysters were held at higher densities during periodic feeding (0.25 oysters L^−1^). All tanks and containers were constantly aerated and supplied with 15.5 °C, and sea water was filtered through a 125-µm filter, through pressurised sand filters, sterilised with UV, and run through a protein skimmer.

There were no significant differences in total wet mass (Iso: 102 ± 4.9 g, Mix: 94.2 ± 4.4 g, Nan: 101 ± 5.8 g; ANOVA *P* = 0.499) or total valve length (Iso: 78.1 ± 1.7 mm, Mix: 75.2 ± 1.4 mm, Nan: 73.4 ± 2.4 mm; ANOVA *P* = 0.185) between diet treatments.

Sea water was routinely monitored to ensure water quality remained within accepted parameters of ammonia (0 ppm), nitrate (2.0–25.0 pp), nitrite (0 ppm), pH (7.8–8.5), salinity (30–33 ppt), and dissolved oxygen (82%–112% saturation). The oysters were fed a mix of microalgae (*Isochrysis galbana* (PLY#565)*, Nannochloropsis gaditana* (CCAP 859/5) and *N. oculata* (CCAP 849, donated by the University of Portsmouth)*, Tetraselmis suecica* (CCAP 66/4)*,* and *Tisochrysis lutea* (CCAP 927/14) during the acclimation period, and each oyster was weighed (g), and total valve length measured (mm). *I. galbana* and a bi-specific combination of *N. gaditana* with *N. oculata* were the species used for the experimental feeding. Both species were cultured in 20 L volumes in Walne Medium in the aquarium, and maintained at consistent 43.5 µmol m^−2^ s^−1^ irradiance, and at 20 °C ± 1 °C (SEM).

Microalgal cell density for feeding was standardised by adapting cell densities used in the literature. A study by Hendriks et al. (2003) fed oyster larvae 1.31 × 10⁷ cells of *I. galbana* per oyster, standardised from calculating microalgal cells for larvae per μm valve length, to account for the increase to average adult valve length of oysters in this experiment. Additional diet calibration was adapted from a study by Pennarun et al. (2003), where 400 adult oysters were fed 2 × 10⁹ cells of *S. costatum* and 8 × 10⁹ cells of *T. isochrysis* (T-ISO) per oyster per day. Based on these parameters, a total density of 1.5 × 10⁸ cells per feed day per container was given. The larval study provided a methodological framework for calculating feed quantities based on oyster size, rather than relying solely on reported concentrations. This approach enabled replication tailored to *M. gigas* adults being fed *I. galbana* and *Nannochloropsis*, ensuring feed volumes were biologically relevant and size appropriate.

Cell counts were performed using a 0.1 mm-deep Improved Neubauer Haemocytometer with Bright Field microscopy, and the relevant volume of microalgae fed to oysters to achieve 1.5 × 10^8^ cells mL^−1^ for each diet condition. The mixed species diet comprised equal cell densities of *I. galbana* and the *Nannochloropsis* spp. Oysters were fed their respective diet three times per week for 11 weeks, with 24 oysters per diet condition for *I. galbana* and the mixed diets, and 23 oysters in the *Nannochloropsis* spp. diet (1.38% mortality rate).

### Respirometry

One oyster was placed into each air-tight 535 mL glass container with an SP-PSt3 Oxygen-Sensitive Planar Foil™ attached for the PreSens Fibox 3™ instrument. Each respirometer was connected to a peristaltic pump to create a semi-continuous flow of water past the oyster, and the system was maintained at incubation conditions. Seven oysters were assessed at one time, with one empty container as a control. Calibrations were set using normal sea water aerated for 5 min (O_2_ saturated) and sea water with 1 g sodium thiosulfite per 100 mL (O_2_-free).

Phase angle measurements were taken every 15 min, and oxygen content was measured in mg L^−1^ (ppm) (Gundersen et al. 1998). Oxygen consumption recordings were taken until O_2_ concentrations fell below 80% of the initial concentrations. At this level or below, the oysters reduced their respiration rate to compensate for the declining O_2_ concentrations (Taylor and Brand 1975; Rivera-Ingraham et al. 2013) and were therefore removed from the experimental containers and returned to their respective tanks.

Respiration rates were calculated using the Eq. 1 below, which measures phase angle between excited and emitted light, where the emitted light has an inverse relationship to O_2_ saturation concentration, therefore, acting as a proxy (Klimant and Wolfbeis 1995). Values were corrected to g AFDW of organic tissue.

1 $$RR=\left({C}_{t0}-{C}_{t1}\right)*({V}_{c}-{V}_{a})*({t}_{1}-{t}_{0})$$

where

*RR*: Respiration rate (mg O_2_ min^−1^ g^−1^),
*C*_*t0*_: Oxygen saturation (mg L^−1^) at *t*_0_,
*C*_*t1*_: Oxygen saturation (mg L^−1^) at *t*_1_,
*V*_*c*_: Volume of container (L),
*V*_*a*_: Volume of animal (L),
*t*_*1*_: Start time (minutes),
*t*_*0*_: End time (minutes), and.
*t*_*1*_* – t*_*0*_: Interval between readings (minutes).

Equation 1. Calculation performed by PreSens Fibox 3™ Fiber Optic Meter.

### Microalgal cell clearance and rejection rates

During a normal feeding day, two oysters were placed into 4 L containers filled with 3 L filtered, protein-skimmed sea water, atop a plastic mount, which itself sat on 53 µm mesh. Algal cell densities were calculated, and a volume of 3.16 × 10^7^ cells mL^−1^ was provided to each container. Oysters fed for 4 h, during which any rejected cells exiting the oyster as pseudofaeces were deposited into the mesh and collected using a Pasteur pipette.

After the 4-h feed, algal cell density was measured again, and clearance rate (cells mL^−1^ h^−1^ individual^−1^) was calculated. Pseudofaeces were wet weighed, then dried at 60 °C for 24 h and re-weighed, and finally ashed in a muddle furnace at 450 °C for 4 h after which ash-free dry weight (AFDW) was determined.

### Stable isotope ratio mass spectrometry (SIRMS) analysis

Following feeding of a mixed diet, it was not possible to ascertain an accurate cell density of individual species of microalgae that remained; the rejected cells (pseudofaeces) homogenised into one mass. Stable isotope ratio mass spectrometry (SIRMS) analysis allows pseudofaeces, or gut contents, to be assessed chemically and elucidate whether oysters preferentially select one species of microalgae when presented with two simultaneously. To determine whether SI composition varied between the two microalgal species, initially 50 mL of *I. galbana* and *Nannochloropsis* spp. (both densities were 6.53 × 10^5^ cells mL^−1^) were collected, pelleted by centrifugation (12,000 × *g* for 5 min) and lyophilised to collect 1 mg of dried microalgae for each species. Samples were analysed in triplicate using a vario PYRO cube elemental analyser (CNS mode) coupled with visION isotope ratio mass spectrometer to measure abundance of C and N, δ^15^N (Air), and δ^13^C (Vienna Pee Dee Belemnite).

Digestive tracts from oysters fed a mixed species diet, containing microalgae from their final feed, and gill samples were also analysed. The gill tissue acted as the background tissue SI reading to correct for oyster tissue SI. Correcting for the oysters’ own tissues allows only the gut contents to be compared to determine if one species of microalga was more abundant than the other.

### Oyster Condition Index (OCI)

At time of oyster dissection, all organic tissues aside from those being used for gene expression analysis were thoroughly removed from the shell cavity. Both the tissues and the shell from each oyster were weighed, dried at 60 °C for 24 h and re-weighed to obtain dry weights. The OCI was calculated using the Eq. 2 (Walne and Mann 1975).

2 $$\text{OCI}=\text{ DTW}* \frac{100}{\text{DSW}}$$

where

OCI: Oyster Condition Index,
DTW: Dry tissue weight (g), and.
DSW: Dry shell weight (g).

Equation 2 Equation to calculate Oyster Condition Index (Walne and Mann 1975).

### Gene expression

Gill tissues were removed from each oyster (approximately 500 µg per sample) and flash frozen in liquid nitrogen. Total RNA was extracted using the TRI Reagent™ protocol (Sigma Aldrich, technical bulletin MB-205). DNA was removed using DNA-Free™ DNase treatment agents (Ambion by Life Technologies, publication 1906 M revision E). cDNA was created using Invitrogen™ SuperScript™ III First-Strand Synthesis system (protocol 18,080.pps) with oligodT priming. RT-qPCR was performed with 25 µL reaction volumes with SYBRgreen dye using the LightCycler 96 (Roche™) with hot start at 90 °C and 30 cycles from 60 to 90 °C, followed by a melt curve to end. Each sample was run in duplicate for each of the eight endogenous reference genes (ERGs) and seven genes of interest (GOIs) listed in Table 1.

The six ERGs are highly conserved and have functions ranging from cell motility (*Cg_β-Actin*) to glycolytic catalysation (*Cg_GAPDH*) and are required for maintenance of basic cellular function. The *a priori *genes of interest were selected based on their respective functions within the oyster biomineralisation pathway (Table 1).

**Table 1:** Overview of putative functions of each of the genes of interest quantified in this study

Gene abbreviation	GenBank accession	Putative function
*Cg_CaM*	KM115543	Regulation of uptake, transport, and secretion of calcium in shell formation
*Cg_EEF1α*	AB122066	Delivery of most aminoacyl-tRNAs to the ribsome
*Cg_Fas*	NC_047562: 29,625,447–29,627,375	Type 1 transmembrane glycoprotein involved in apoptosis induction
*Cg_GAPDH*	AJ544886	Catalyses sixth step of glycolysis, converting glyceraldehyde 3-phosphate to D-glycerate 1,3-biphosphate
*Cg_MG4*	AAZ76258	Calcium-binding protein responsible for nucleation of calcium compounds
*Cg_Nac*	NM_001305309	Carbonic anhydrase domain nacrein-like protein related to the aragonitic nacre layer of molluscan shells
*Cg_Perl*	P82596	Facilitates CaCO_3_ nucleation and crystal growth in shell formation
*Cg_Tub*	CB617442	Polymerises filaments that form microtubules that act as cellular skeletons
*Cg_Ube2g1*	XM_01143986	Links activated ubiquitin via a transthiolation reaction in ubiquitylation
*Cg_β-Act*	AF172606	Ubiquitously, highly expressed protein involved in cell motility and muscle contraction

RT-qPCR output was analysed in Biogazelle qbasePLUS™ using relative cNRQ values calculated from amplification data. Following geNorm analysis, six of the eight ERGs were of sufficient stability to be used to calibrate GOI cNRQ data.

### Statistical analyses

All statistical tests were performed in RStudio (v. 4.2.2.). Normality was tested using Shapiro–Wilk (S-W). Normally distributed data (*P* > 0.05 S-W result) were then analysed using the one-way ANOVA with Tukey’s post hoc test (if required), and significantly skewed data (*P* < 0.05 S-W result) were assessed for significance using the Kruskal–Wallis test with Šídák correction and Dunn’s post hoc test (if required).

## Results

### Respiration rates

**Fig. 2:**
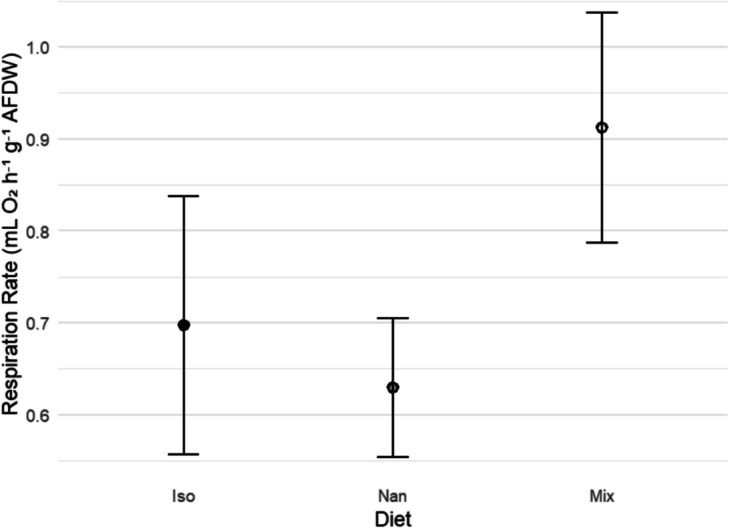
Average respiration rate of oysters between the three diet conditions. No significant differences (ANOVA *F* = 0.054, *P* = 0.947, *n* = 71) were recorded between the respiration rates (RR) (mg O_2_ h^−1^ g^−^.^1^ AFDW) ± SEM for oysters fed each of the three diet conditions *Isochrysis galbana* (Iso), *Nannochloropsis* spp. (Nan), and mixed diet (Mix)

The respiration rate of oysters (Fig. 2) fed *I. galbana* was 12.63 (± 4.44 SEM) mg O_2_ h^−1^ gAFDW^−1^ (*n* = 24), whilst *Nannochloropsis* spp.-fed oysters’ respiration rate was 13.21 (± 3.16 SEM) mg O_2_ h^−1^ gAFDW^−1^ (*n* = 23), and mixed diet oysters’ respiration rate was 14.24 (± 2.74 SEM) mg O_2_ h^−1^ gAFDW^−1^ (*n* = 24). There were no significant differences in respiration rates between diet conditions when tested with one-way analysis of variance (ANOVA) (*F* = 0.05412, *P* = 0.947, *n* = 71).

### Cell clearance and rejection rates

**Fig. 3:**
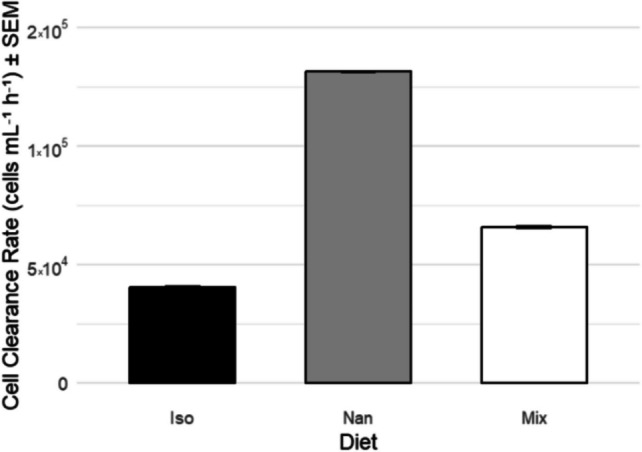
Average cell clearance (cells mL^−1^ h^−1^ gAFDW^−^.^1^) ± SEM. No significant differences were identified through one-way ANOVA (*F* = 0.263,* P* = 0.770,* n* = 71)

Cell clearance rates (Fig. 3) showed that on average, oysters fed *I. galbana* cleared 4.98 × 10^7^ cells mL^−1^ h^−1^ gAFDW^−1^ (± 1.55 × 10^7^ SEM) cells per individual, those fed *Nannochloropsis* cleared 5.32 × 10^7^ cells mL^−1^ h^−1^ gAFDW^−1^ (± 9.66 × 10^6^ SEM) cells per individual, and those fed mixed species cleared 4.24 × 10^7^ cells mL^−1^ h^−1^ gAFDW^−1^ (± 3.70 × 10^6^ SEM). One-way ANOVA showed no significant difference between all means (*P* = 0.77,* n* = 71).

The total AFDW of pseudofaeces produced by all 71 oysters totalled 288.7 mg. Mixed diet oysters produced the most, with seven oysters producing 0.719 g AFDW^−1^ (± 0.266 SEM) in 4 h. One *Nannochloropsis* spp.-fed oyster produced 1.193 g AFDW^−1^, and no *I. galbana* condition oysters produced any pseudofaeces. These values have been corrected for AFDW of the oyster.

**Fig. 4:**
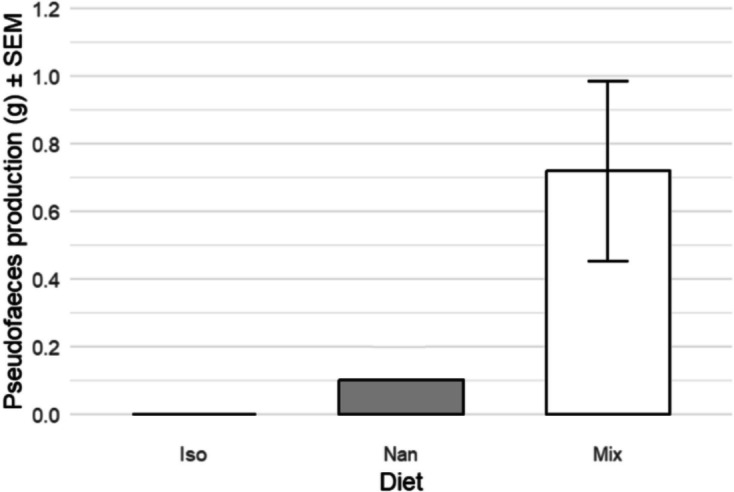
Average cell rejection in ash-free dry weight (AFDW) of pseudofaeces (pf) ± SEM. Means of pseudofaeces production by oysters fed each of the three diet conditions were significantly different, tested by Kruskal–Wallis (*H* = 10.815*, P* = 0.00448, *n* = 72) with Dunn’s post hoc showing mixed diet-fed oysters produced significantly more than those fed *I. galbana* (*Z* = 3.057, *P* = 0.00224**, *n* = 48) and *Nannochloropsis* spp. (*Z* = 2.580, *P* = 0.0099**, *n* = 47)

Kruskal–Wallis testing showed that the pseudofaeces production of oysters fed different diets was significantly different from one another (*H* = 10.815, *P* = 0.00448, *n* = 72), where Tukey’s post hoc testing showed mixed diet-fed oysters producing much more pseudofaeces than the other oysters (*I. galbana*/*Nannochloropsis* spp. *Z* = 0.477, *P* = 0.634, *n* = 47; *I. galbana*/mixed species (*P* = 0.00224**, *n* = 48; *Nannochloropsis* spp./mixed species (*P* = 0.0099**, *n* = 47) (Fig. 4).

### *Differences in δ*^*15*^*N and δ*^*13*^*C signatures of microalgae in oyster digestive tract*

**Fig. 5:**
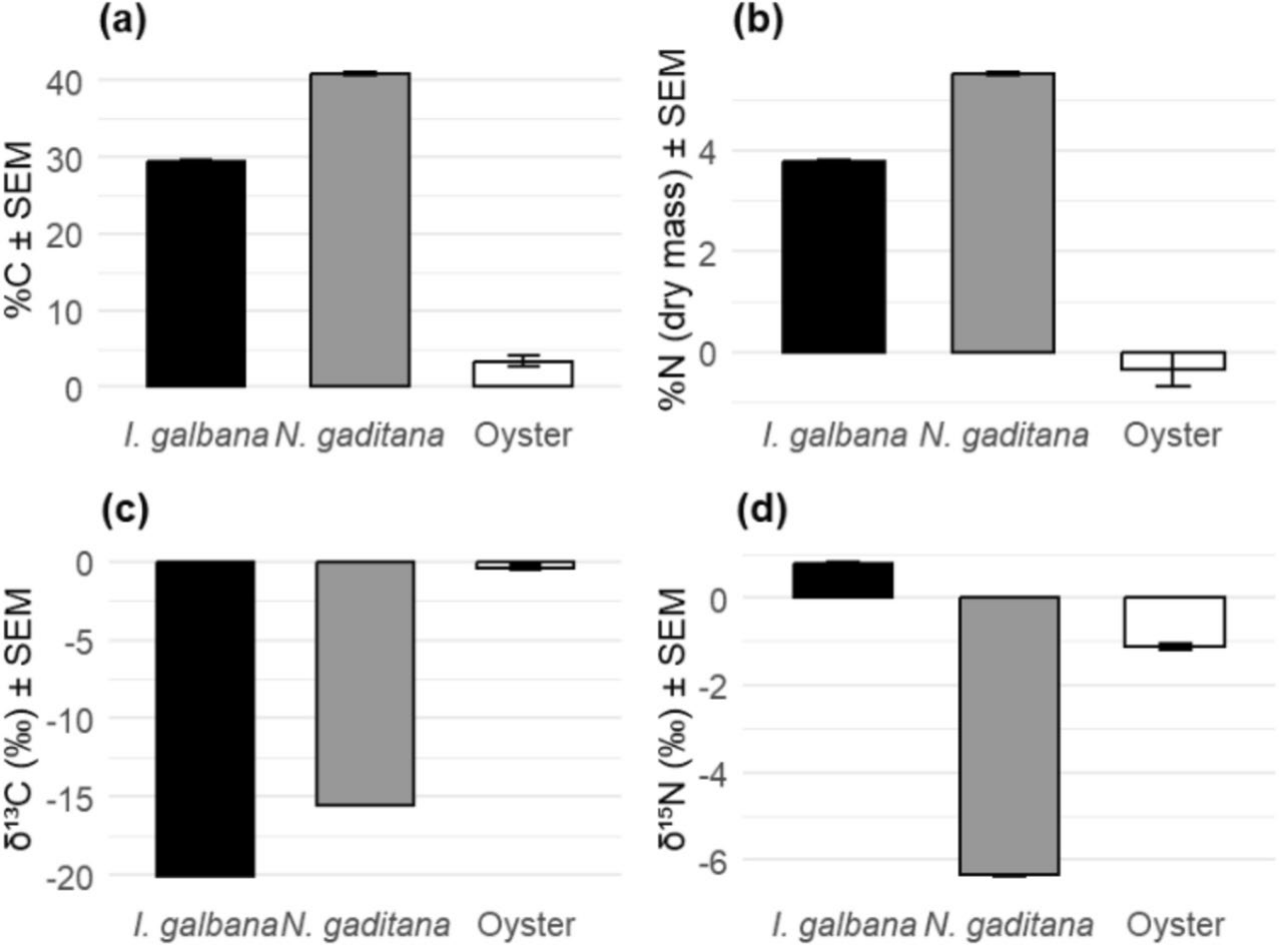
Elemental composition and stable isotope ratios (mean ± SEM) in *I. galbana, N. gaditana,* and oyster digestive tracts. **a** Percentage of carbon (%C), **b** percentage of nitrogen (%N, dry mass), **c** stable carbon isotopic composition (δ^13^C, ‰), and **d** stable nitrogen isotopic composition (δ^15^N, ‰). Note: *N. oculata* was not available at the time of analysis; therefore, SIRMS was conducted using *N. gaditana* only

Despite both *I. galbana* and *Nannochloropsis* spp. using the C3 photosynthetic system, and therefore the same mechanisms of processing nitrogen (Urban et al. 2021), the stable isotopic composition greatly varied between both (Table 3 and Fig. 5) allowing the different species to be identified in the oyster gut. An unpaired *t*-test showed that the mean abundance (% for elemental abundance, ‰ for isotope ratios) of all SI’s was significantly different between *I. galbana* and *N. gaditana*.

The average elemental and isotope values for the contents of digestive tracts (dt) were 0.42% N, 3.92% C, − 1.12 ‰ δ^15^N, − 0.38 ‰ δ^13^C. These SI abundance means were compared to those of each microalgae species to determine whether oysters would show a preference in clearing a particular species. A Welch’s unpaired *t*-test for unequal sample sizes showed that SI abundance in oyster digestive tracts differed significantly to those of both microalgae (Table 2). This suggests that both species of microalgae were consumed and therefore effect size between the microalgal SI and the oyster dt SI was analysed with the Cohen’s *d* test to quantitatively measure the magnitude of the relationship between each microalga in the digestive tract. Cohen’s *d* analysis has been used frequently in predator gut analyses to shed light on tropic interactions and prey selection, for example in insectivorous birds (Razeng and Watson 2015) and rattlesnakes (Dugan and Hayes 2012) and has been used to discern host fitness in *Daphnia* gut microbiome assemblies (Gurung et al. 2024).

**Table 2:** Results of Welch’s unequal sample size *t*-test of oyster digestive tract (dt) SI abundance (‰) and that of *I. galbana* and *N. gaditana*

	*I. galbana* and oyster digestive tract				*N. gaditana* and oyster digestive tract			
SI	*t*	*P*	*df*	*n*	*t*	*P*	*df*	*n*
N%	− 12.22	< 0.0001	20.28	24	− 17.35	< 0.0001	20.28	24
C%	− 31.75	< 0.0001	20.09	24	− 48.88	< 0.0001	21.88	24
δ^15^N	− 21.12	< 0.0001	19.22	24	59.68	< 0.0001	20.94	24
δ^13^C	107.11	< 0.0001	21.78	24	86.31	< 0.0001	21.30	24

To account for background isotopic variation, digestive tissue values were normalised by subtracting corresponding gill tissue measurements from each individual. Negative values indicate that, on average, the digestive tissue contained lower elemental or isotopic concentrations than the gill, likely reflecting differences in metabolic activity or assimilation dynamics following feeding.

All effect sizes were large based on classification by Cohen (1988) (Table 3); however, *I. galbana* were considerably smaller across three of the four SI’s, showing that there was a strong correlation between the microalgal contents of the digestive tracts and *I. galbana*, which suggested that they consumed more *I. galbana* than *Nannochloropsis* when offered both simultaneously, in equal cell densities.

**Table 3:** Cohen’s *d* effect size results between stable isotopes (SI) in *I. galbana* and oyster digestive tracts (dt) and in *N. gaditana* and oyster dt. Larger numbers indicate a weaker relationship between the variables, and smaller numbers indicate a stronger relationship

	*I. galbana* and oyster digestive tract	*N. gaditana* and oyster digestive tract
SI	Cohen’s *d*	Cohen’s *d*
N%	3.78	5.37
C%	10.65	15.56
δ^15^N	7.16	19.78
δ^13^C	34.89	27.10

### Changes in oyster mass

**Fig. 6:**
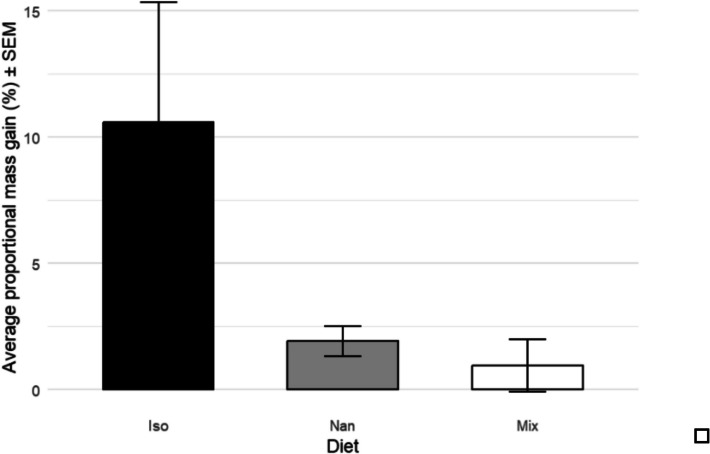
Proportional mass increase (%), before and after the 11-week feeding experiment, analysed with Kruskal–Wallis (*H* = 9.8547, *P* = 0.00725**, *n* = 71) with Dunn’s post hoc testing between the three diet conditions (*I. galbana*/*Nannochloropsis* spp. *Z* = 0.407, *P* = 0.684, *n* = 47; *I. galbana*/mixed *Z* = 2.9065, *P* = 0.00365**, *n* = 48; *Nannochloropsis* spp./mixed *Z* = 2.4685, *P* = 0.0136*, *n* = 47)

The average total mass increases (Fig. 6) were 5.08 g, 1.61 g, and 0.75 g for oysters fed *I. galbana*, *Nannochloropsis* spp., and mixed diet respectively. Proportionally, oysters fed *I. galbana* gained 10.58% mass (± 4.8%), those fed *Nannochloropsis* spp. gained 1.92% mass (± 0.6%), and those fed the mixed species diet gained 0.96% mass (± 1.04%) (Fig. 5). Kruskal–Wallis analysis showed a significant difference between proportional weight gain (*H* = 9.8547, *P* = 0.00725**, *n* = 71). Dunn’s post hoc testing showed that those fed the mixed species diet gained significantly less proportional mass than those fed the other diets (*I. galbana*/*Nannochloropsis* spp. *P* = 0.684, *n* = 47; *I. galbana*/mixed *P* = 0.00365**, *n* = 48; *Nannochloropsis* spp./mixed *P* = 0.0136*, *n* = 47).

### Oyster condition indices

**Fig. 7:**
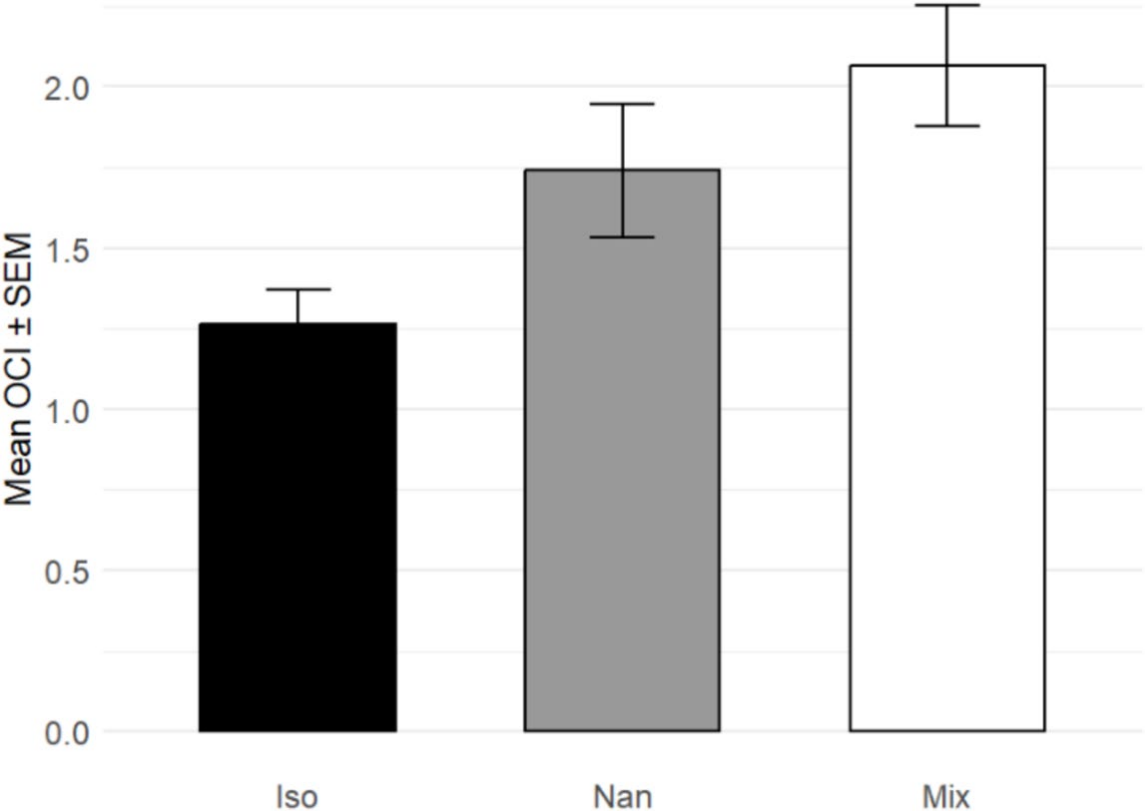
Average Oyster Condition Index (OCI) value with SEM. As data were not normally distributed, Kruskal–Wallis with Sidak correction test was used, showing a significant difference between the means of all three OCI’s (*H* = 3.595, *P* = 0.037, *n* = 71). Dunn’s post hoc test revealed *I. galbana*-fed oyster OCIs were significantly lower than those of oysters fed *Nannochloropsis* spp. (*Z* = 2.1384, *P* = 0.033, *n* = 47), or a mixed species diet (*Z* = 2.2938, *P* = 0.022, *n* = 48)

The average OCI (Fig. 7) of *I. galbana* diet oysters was 0.640 (± 0.04), *Nannochloropsis* spp. diet oysters were 0.811 (± 0.06), and mixed oysters are 0.806 (± 0.05). Kruskal–Wallis testing showed that the OCI’s were significantly different from one another (*P* = 0.0007, *n* = 71). Dunn’s post hoc testing showed that oysters fed *I. galbana* had a significantly lower OCI than those fed *Nannochloropsis* spp. (*P* = 0.036, *n* = 47) or mixed species (*P* = 0.0008, *n* = 48). There was no significant difference between OCIs of oysters fed *Nannochloropsis* spp. and a mixed species diet (*P* = 0.22, *n* = 47). The OCI provides a ratio of dry tissue to dry shell weight, indicating *I. galbana*-fed oysters invested comparatively more into the growth of their organic tissues than their mineral shells.

### Regulation of biomineralisation gene expression

**Fig. 8:**
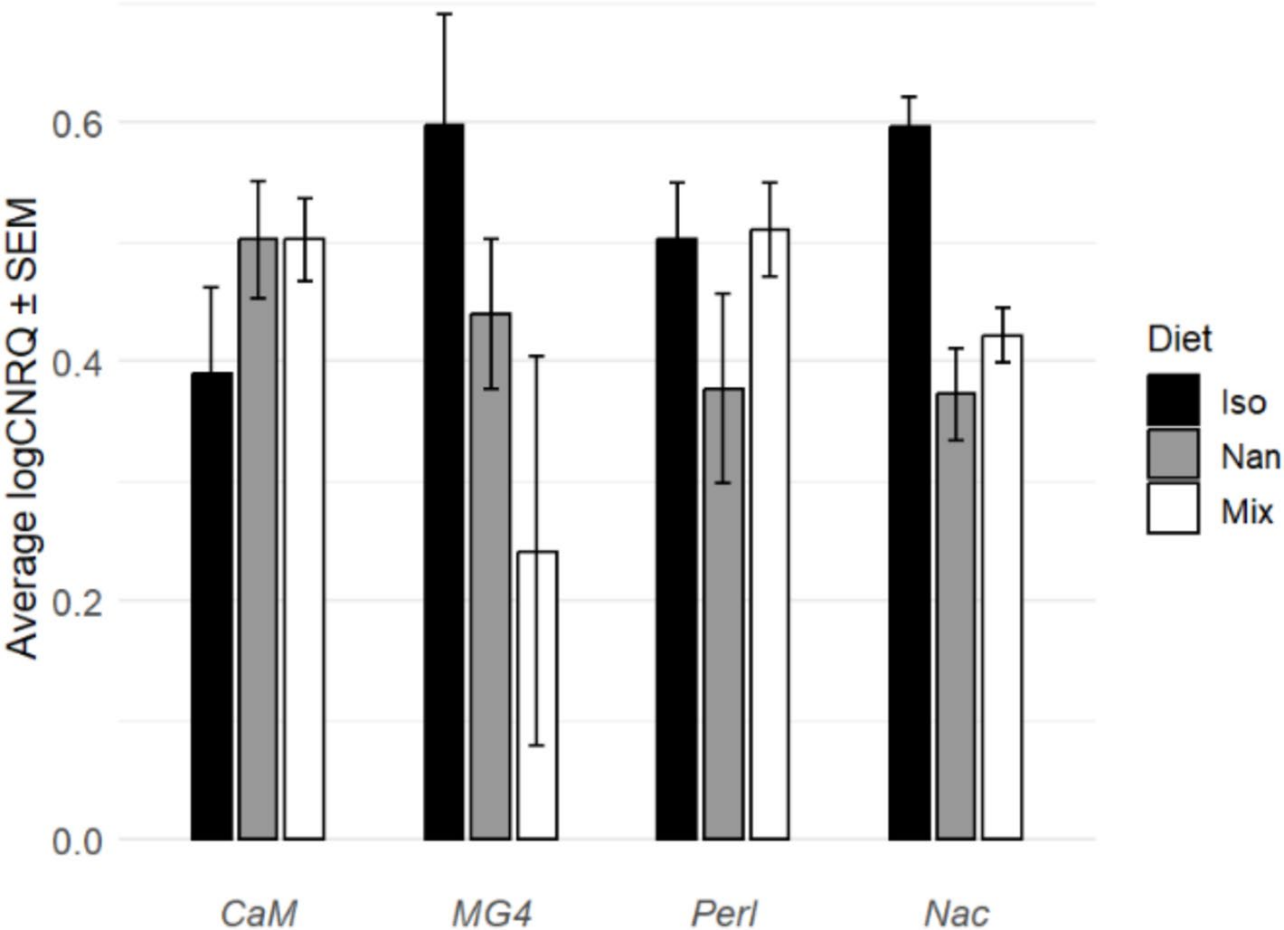
Relative log transformed cNRQ values with SEM for all genes of interest. *CaM* = calmodulin, *MG4* = mantle gene 4, *Perl* = perlucin, *Nac* = nacrein. One-way ANOVA with Tukey’s post hoc test showed a significant upregulation in the expression of *Nac* in gill tissues of oysters fed *I. galbana* (*F* = 16.146, *P* = 0.00015, *n* = 12) compared to those fed *Nannochloropsis* spp. (*Q* = 7.643, *P* = 0.0011 *n* = 8) and a mixed species diet (*Q* = 5.972, *P* = 0.0057, *n* = 8). No other genes were significantly up- or downregulated

For three of the four genes of interest (*CaM*, *MG4*, and perlucin), no significant differences in expression between diet species were identified (Fig. 8). However, the calcification gene nacrein was significantly upregulated in *I. galbana* diet oysters compared to the other two diet condition oysters when analysed with one-way ANOVA (*P* = 0.001, *n* = 12). This is in line with the previous findings that *I. galbana*-fed oysters do not grow shell at the same rate as oysters fed *Nannochloropsis* spp. or a mixed species diet.

## Discussion

This research aimed to elucidate physiological responses of *M. gigas* (metabolism, cell clearance and rejection rates, total wet mass and OCI, and regulation of biomineralisation gene expression) when fed *I. galbana, Nannochloropsis* spp., or a mixed diet. It was shown that Pacific oysters initially select *I. galbana* cells in a mixed-algae diet, increase biomass, and expression of biomineralisation gene nacrein.

Although continuous feeding would have been ideal to better mimic natural filtration behaviour, this was not feasible due to the lack of available resources for continuous delivery systems or real-time enumeration of algal cells to monitor densities. As such, oysters were fed on the same schedule as general University protocols, which may have introduced fasting periods between feedings. These could potentially influence filtration behaviour and growth and should be considered when interpreting the results.

The duration of the experiment (11 weeks) was intentionally shorter than a full production cycle (18 to 30 months) or the species’ lifespan (over 5 years). This decision was based on prior in-lab findings where gene expression changes were observable after just 5 weeks. The aim here was to establish a physiological ‘baseline’ for adult *M. gigas* when fed three different diets. Interestingly, the observed changes in stable isotope ratios and nacrein expression were not the original focus but emerged as valuable outcomes, guiding the study towards a deeper exploration of biomineralisation.

Whilst this experiment focused on adult oysters, juveniles were used in a subsequent study to explore developmental stage-specific responses. The emphasis on the relationship between single-species diets and biomineralisation evolved from unexpected findings in organic content index (OCI) results. Initially, the study was not designed to investigate shell formation specifically, but the project’s progression naturally led to this focus.

### Metabolic rate, clearance and rejection rates, and gut SI composition

Recent studies have demonstrated the physiological importance of *I. galbana* in adult Pacific oysters, particularly in relation to feeding efficiency and metabolic performance. Cyrille et al. (2020) established reference intervals for clearance and oxygen consumption rates in adult *M. gigas* under controlled conditions, using *I. galbana* as the sole dietary input. Their findings showed that oysters maintained on *I. galbana* exhibited clearance rates between 0.7 and 4.1 L h^−1^ g AFDW^−1^ and oxygen consumption rates of 0.4 and 1.3 mg O₂ h^−1^ g DW^−1^, highlighting its suitability as a benchmark feed species for physiological assessments. Similarly, Nielsen et al. (2017) found that adults retained *I. galbana* with slightly reduced efficiency compared to larger algal species, yet still achieved high clearance rates, suggesting that *I. galbana* remains a valuable dietary component even in adults.

The full nutritional profile of *I. galbana* and other common aquaculture feed species (for example *Nannochloropsis gaditana, Tetraselmis suecica, Chaetoceros calcitrans,* and *Thalassiosira pseudonana*) is well established. The carbohydrate, lipid, protein, and essential amino acid content of microalgae has been described for all major Classes. Haptophytes have slightly higher lipid, carbohydrate, isoleucine, leucine, methionine, tryptophan, and valine content than eustigmatophytes, and eustigmatophytes have higher protein, histidine, proline, and threonine (Brown et al. 1997).

The respiration rates between oysters fed single and mixed species diets were not significantly different, indicating that both *I. galbana* and *Nannochloropsis* spp. provide sufficient nutritional resources to meet the metabolic needs of adult *M. gigas*, and therefore, the metabolic rate was not affected by the diets tested. These results also show that the allocation of energy to organic tissue growth in *I. galbana*-fed oysters and the investment in shell growth in *Nannochloropsis* spp.-fed oysters do not directly correspond to differences in metabolic rate. As an intertidal species, oysters can adapt to low-oxygen environments when tides recede. Rather than using oxygen, oysters will rely on stored carbohydrates, resulting in metabolic depression (Corporeau et al. 2022), and as the oysters used in this study were not significantly different in starting or ending wet mass (total, including shell), nor in AFDW of meat alone, the difference in metabolic rate was not anticipated.

The most interesting finding of this study is the preferential selection of *I. galbana* by *M. gigas* when presented with an equal mix of *Nannochloropsis gaditana* and *oculata*, and *I. galbana*. Stable isotopes of animal tissue are largely determined by the SI composition of their diet, though other biotic and abiotic factors also contribute. It can also be influenced by dissolved oxygen concentration (Wassenaar et al. 2024) and temperature (Shipley and Matich 2020). Despite the unpaired *t*-test results being non-significant, the Cohen’s *d* values showed substantial effect sizes for both *I. galbana* (N% = 3.78, C% = 10.65, δ^15^N = 7.16, δ^13^C = 34.89) and *N. gaditana* (N% = 5.37, C% = 15.56, δ^15^N = 19.78, δ^13^C = 27.10), suggesting that oysters consumed both microalgae species (as opposed to only consuming one and completely rejecting the other). However, the stronger preference for *I. galbana* was evident from its lower Cohen’s *d* values, highlighting the oysters’ dietary preference in a way that traditional significance tests did not capture.

Similar results highlighting significant differences in clearance rate between diet conditions in bivalves, where the sensory impact of mixed diet treatments resulted in significantly higher clearance rates (Vanderploeg et al. 2001). Additionally, two studies by Liu et al. (2009; 2014) showed that when offered a mixed diet, cell clearance rates are almost double the single-species diet clearance rates. Whilst in these cases, particle size was deemed to be the most likely factor influencing clearance rates, further investigation is warranted to elucidate the underlying mechanisms governing these dietary preferences.

This preference may be attributed to various factors, including particle size, composition, and nutritional content. The average particle size of *Isochrysis* is 5 to 6 μm (Meneses-Montero et al. 2025), and *Nannochloropsis* cells are 2 to 5 μm (Baroni et al. 2019), and geometrically, *Isochrysis* are rounded in shape, and *Nannochloropsis* are slightly elongated. *Nannochloropsis* species tend to aggregate in pairs due to polysaccharide secretion (Morales-Plasencia et al. 2023) which could potentially negate any size-related selection, making it plausible that oysters are selectively clearing *I. galbana* first based on preferences such as nutritional composition or shape, something that requires further investigation.

### Changes in mass, Oyster Condition Index, and nacrein expression

The observed decrease in OCI for oysters fed single species *I. galbana* compared to those fed single species *Nannochloropsis* spp. or a mixed diet of both species is significant. This result indicates that oysters consuming *I. galbana* invested significantly less in shell growth, diverting their resources towards internal tissue growth, supported by the changes in gene expression recorded in this study. The upregulation of nacrein usually occurs after the oyster enters the spat stage (Song et al. 2014). Nacrein is most abundantly found in mantle tissues, followed by gill tissues, but is also prevalent in the gonad, gut, adductor muscle, and haematocytes (Song et al. 2022).

Nacrein is a carbonic anhydrase domain protein which regulates calcification of the nacreous layer of bivalve shells. It has been shown to have both precipitatory and inhibitory effects; supporting biomineralisation through converting CO_2_ to HCO_3_^−^ to provide bicarbonate ions (Sharker et al. 2021), and also by inhibiting precipitation of CaCO_3_ in the extrapallial space between the oysters’ shell and mantle organ (Miyamoto et al., 2005). In *M. gigas,* two new nacrein-like proteins (F1 and F2) were described (Song et al. 2014), and research into its role in oyster shell physiology has shown that nacrein activity is decreased when the NF-κB signalling pathway is inhibited (Sun et al. 2015).

Another plausible theory that could explain the significant upregulation of nacrein, as well as the noticeably higher MG4 expression (though not significant), is that the large increase in tissue mass following the *I. galbana* diet (Fig. 6) required a proportional increase in shell formation. Such a shift in resource allocation could be particularly beneficial for aquaculture practices, as the edible part of the oyster (the soft tissues) is the primary product of interest, whilst current pricing practices are based on total oyster mass including the shell. This aligns with commercial norms across major oyster-producing regions, where oysters are typically sold by wet weight (including shell) when live or in-shell, as reported by the FAO and national trade bodies (FAO 2021a, b; NOAA 2023). In the UK and EU, oysters are commonly sold by count or wet weight, and in the US, trade classifications distinguish between live (wet weight) and shucked (drained weight) oysters. These findings underscore the potential for optimising feeding strategies in oyster cultivation to enhance the yield of desired oyster products, particularly if pricing models evolve to better reflect tissue yield.

A mixed diet including *I. galbana* is thought to be optimal in oyster aquaculture, as what is nutritionally lacking from the prymnesiophyte can be compensated with other species of microalgae (Rico-Villa et al. 2006; Ronquillo et al. 2012). However, this study has shown that a single species diet comprising just *I. galbana* can have significantly positive effects on oyster growth, and they will selectively consume *I. galbana* when presented with a simultaneous choice.

### Wider implications for Pacific oyster aquaculture

Whilst oysters are most often cultured in crates on the seabed, controlling microalgal diet would be difficult. However, being able to biologically manipulate shell formation at different stages of oyster development can affect their likelihood of survival. For example, if it were possible to increase shell growth immediately post-settlement but before grow-out, the oysters would be better protected in terms of predation risk. At advanced stages, in preparation for harvest, altering diet to increase tissue growth might improve the value of the product as there would be more edible content, though investigation into the integrity of the shell and how the reallocation of growth would be needed to ensure that the aesthetic appeal of the oyster shells is maintained for industry. These insights can be used to develop stage-specific feeding strategies that optimise oyster growth and market readiness, ultimately contributing to more sustainable and profitable aquaculture practices.

## Data Availability

All raw data collected is publicly available on the Cefas Data Portal at 10.14466/CefasDataHub.164.
